# A high‐resolution (1.2 Å) crystal structure of the anti‐CRISPR protein AcrIF9

**DOI:** 10.1002/2211-5463.12986

**Published:** 2020-11-05

**Authors:** Gi Eob Kim, So Yeon Lee, Hyun Ho Park

**Affiliations:** ^1^ Department of Global Innovative Drugs Graduate School of Chung‐Ang University Seoul Korea; ^2^ College of Pharmacy Chung‐Ang University Seoul Korea

**Keywords:** AcrIF9, adaptive immunity, anti‐CRISPR proteins, CRISPR‐Cas system, crystal structure

## Abstract

Prokaryotic adaptive immunity by CRISPR‐Cas systems, which confer resistance to foreign genetic elements, has been used by bacteria to combat viruses. To cope, viruses evolved multiple anti‐CRISPR proteins, which can inhibit system function through various mechanisms. Although the structures and mechanisms of several anti‐CRISPR proteins have been elucidated, those of the AcrIF9 family have not yet been identified. To understand the molecular basis underlying AcrIF9 anti‐CRISPR function, we determined the 1.2 Å crystal structure of AcrIF9. Structural and biochemical studies showed that AcrIF9 exists in monomeric form in solution and can directly interact with DNA using a positively charged cleft. Based on analysis of the structure, we suggest part of the anti‐CRISPR molecular mechanism by AcrIF9.

## Graphical abstract

This study determined the 1.2 Å high‐resolution crystal structure of AcrIF9 for the purpose of understanding the molecular basis underlying its anti‐CRISPR function. This high‐resolution crystal structure was compared with the identified structure of the cryo‐electron microscopy structure of AcrIF9 associated with the cascade complex to analyze feature similarities and differences.

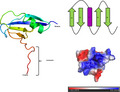

**Abbreviations**
Acr: anti‐CRISPR proteins
Cas: CRISPR‐associated protein
CRISPRs: Clustered regularly interspaced short palindromic repeats
EM: electron microscopy
FPLC: fast protein liquid chromatography
MALS: multi‐angle light scattering
SEC: size‐exclusion chromatography

Clustered regularly interspaced short palindromic repeats (CRISPRs), which are repetitive DNA sequences found in the genomes of prokaryotic organisms such as bacteria and archaea, are derived from the DNA fragments of invaders, such as bacteriophages (bacterial viruses), that previously infected those organisms [1, 2, 3]. Because prokaryotes use these CRISPRs to detect and destroy DNA from similar bacteriophages during subsequent infections, CRISPRs play a critical function in the antiphage defense system of prokaryotes by constructing adaptive immunity [1, 4, 5].

CRISPR‐associated protein (Cas) is an enzyme that recognizes and destroys specific DNA strands that are complementary to the CRISPR sequence [6]. For proper function of CRISPR‐Cas systems, one or more Cas proteins form a complex with a small RNA fragment called trans‐activating crispr RNA (crRNA), which is transcribed and processed from the host CRISPR sequence [7, 8]. This Cas protein–crRNA complex can target and cleave DNA or RNA from the invaders with a sequence complementary to the crRNA [7, 8]. Because CRISPR‐Cas systems can cleave the desired DNA sequence, they can be used to edit genes within organisms for various applications in basic biological research and disease treatments [9, 10].

CRISPR‐Cas systems can be classified into two broad classes, class 1 and class 2, which encompass six types (type I to type VI) based on their action mechanisms [5, 8]. Class 1 systems, including types I, III, and IV, are constructed by multi‐subunit Cas proteins for performing multiple functions, whereas class 2 systems, including types II, V, and VI, use a single huge protein containing all necessary activities to recognize and destroy the target DNA [8, 11].

To counteract this prokaryotic immune system that confers resistance to foreign genetic elements, phages evolved to have multiple anti‐CRISPR genes that encode anti‐CRISPR proteins (Acr) that can inhibit the host CRISPR‐Cas system function [12, 13, 14]. Based on genome searches and comparisons with advanced machine learning tools, approximately 60 Acr genes have been identified thus far [12, 15]. Because Acr genes have low and unrelated sequence homology, they are classified based on the targeted CRISPR‐Cas systems [11, 12].

Although the diverse structures and mechanisms of many Acr have been revealed [11, 14, 16, 17], the inhibitory mechanism of the AcrIF9 family has not been identified due to limited structural information. Thus, to understand the molecular basis underlying the AcrIF9 anti‐CRISPR function, we determined the 1.2 Å high‐resolution crystal structure of AcrIF9. Structural and biochemical studies showed that AcrIF9 exists in monomeric form in solution and can directly interact with DNA using a positively charged cleft. During our manuscript preparation, the cryo‐electron microscopy (EM) structure of AcrIF9 associated with the cascade complex was released [18]. Based on comparisons with the structure of cascade‐complexed AcrIF9, we identified a number of similarities and differences in various features of the AcrIF9 structure.

## Materials and methods

### Cloning, overexpression, and purification

The AcrIF9 gene from the *Pseudomonas aeruginosa* phage was synthesized by Bionics (Daejeon, Korea) and cloned into a pET21a plasmid vector (Novagen, Madison, WIS, USA) with a C‐terminal polyhistidine tag. The *Nde*I and *Xho*I restriction sites were used for cloning. The procedures and methods used for expression and purification of this target protein were similar with those used for our previous study [19]. The resulting recombinant vector containing the full‐length AcrIF9 (residues 1–68) gene was transformed into *Escherichia coli* strain BL21(DE3) competent cells. The cells were cultured at 37 °C in 1 L of lysogeny broth containing 50 μg·mL^−1^ kanamycin. When the optical density value at 600 nm reached 0.7, the temperature was adjusted to 20 °C, and 0.5 mm isopropyl β‐d‐1‐thiogalactopyranoside was added for induction of the target gene. The induced cells were further cultured for 18 h in a shaking incubator. The cultured cells were harvested by centrifugation at 2000 ***g*** for 15 min at 4 °C, resuspended in lysis buffer [20 mm Tris/HCl (pH 8.0), 500 mm sodium chloride, and 25 mm imidazole], and lysed by ultrasonication at 4 °C. The cell lysate and supernatant were separated by centrifugation at 10 000 ***g*** for 30 min at 4 °C. The collected supernatant was mixed with Ni‐nitrilotriacetic acid (NTA) affinity resins for 3 h, and the mixture was loaded onto a gravity‐flow column (Bio‐Rad, Hercules, CA, USA). To remove impurities, the resin was washed with 50 mL of washing buffer [20 mm Tris/HCl (pH 8.0), 500 mm NaCl, and 60 mm imidazole]. After washing, the resin‐bound target protein was eluted from the resin in the column using elution buffer [20 mm Tris/HCl (pH 8.0), 500 mm NaCl, and 250 mm imidazole]. AcrIF9 was further purified by size‐exclusion chromatography (SEC) using a Superdex 200 10/300 GL column (GE Healthcare, Waukesha, WI, USA), which had been pre‐equilibrated with a solution comprising 20 mm Tris/HCl (pH 8.0) and 150 mm NaCl. The target protein eluted from SEC was collected, pooled, and concentrated to 3.0 mg·mL^−1^ for crystallization. The purity of the protein was visually assessed using SDS/PAGE.

### Multi‐angle light scattering analysis

The absolute molecular weight of AcrIF9 in solution was measured using SEC‐coupled multi‐angle light scattering (SEC‐MALS). The protein solution was loaded onto a Superdex 200 Increase 10/300 GL 24 mL column (GE Healthcare) pre‐equilibrated with an SEC buffer [20 mm Tris/HCl (pH 8.0) and 150 mm NaCl]. The flow rate of the buffer was controlled to 0.4 mL·min^−1^, and SEC‐MALS was performed at 20 °C. A DAWN‐TREOS MALS detector was connected to an ÄKTA Explorer system. The molecular weight of bovine serum albumin was measured for a reference value. Data were processed and assessed using astra software (WYATT technology, SantaBarbara, CA, USA).

### Crystallization and X‐ray diffraction data collection

AcrIF9 was crystallized using the hanging‐drop vapor diffusion method at 20 °C. Initial crystals were obtained by equilibrating a mixture containing 1 μL of protein solution [3.0 mg·mL^−1^ protein in 20 mm Tris/HCl (pH 8.0) and 150 mm NaCl] and 1 μL of a reservoir solution containing 0.1 m Tris/HCl (pH 8.5), 25% (w/v) PEG‐3350, and 0.18 m ammonium acetate against 0.3 mL of reservoir solution. The crystallization conditions were further optimized by experimenting with a range of protein and precipitant concentrations at various pH values. As a result, the best crystals were obtained by adding 4% (v/v) 2,2,2‐trifluoroethanol at the reservoir solution. The optimized crystals appeared in 14 days. A single crystal was selected and soaked in reservoir solution supplemented with 40% (v/v) glycerol for cryoprotection. X‐ray diffraction data were collected at −178 °C on the beamline BL‐5C at the Pohang Accelerator Laboratory (Pohang, Korea). Data processing, including indexing, integration, and scaling, was conducted using hkl2000 software [20].

### Structure determination and refinement

The structure was determined using ARCIMBOLDO_BORGES *ab initio* phasing software [21], combining fragment search with phaser [22] and density modification with shelxe [23]. The initial model was built automatically using AutoBuild from the phenix package, and further model building with refinement was performed using coot [24] and phenix.refine [25]. The full anisotropic refinement was used. The structure quality and stereochemistry were validated using molprobity [26]. All structural figures were generated using the pymol program [27].

## Results and Discussion

### Overall structure of AcrIF9 from the *P. aeruginosa* phage

**Fig. 1:**
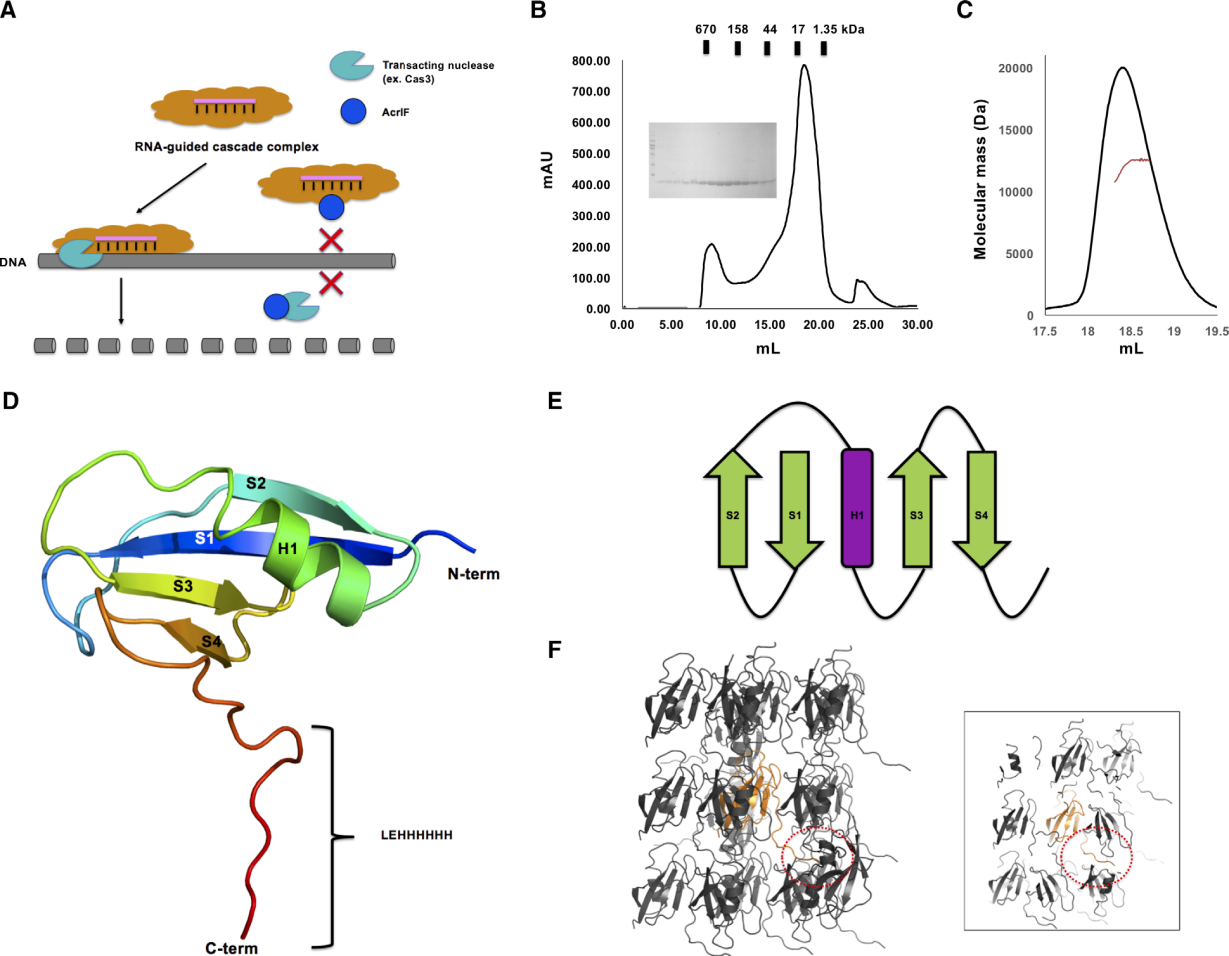
Overall structure of AcrIF9. (A) Overview of the known inhibition mechanisms of type I‐F CRISPR‐Cas by the AcrIF family. (B) SEC profile of AcrIF9. (C) MALS profile of AcrIF9. The experimental MALS data (red line) are plotted as SEC elution volume (*x*‐axis) versus absolute molecular mass (*y*‐axis) distributions on the SEC chromatogram (black) at 280 nm. (D) Cartoon representation of AcrIF9. The color of the chain from the N termini to the C termini gradually moves through the spectrum from blue to red. The four antiparallel β‐sheets and one α‐helix are labeled S1–S4 and H1, respectively. Extra residues from the expression construct (LEHHHHHH) are indicated at the C terminus. (E) Topology representation of AcrIF9. (F) Crystallographic packing of AcrIF9. The single AcrIF9 molecule in the asymmetric unit is colored in orange. The other gray molecules are symmetrical molecules. The C‐terminal six‐histidine tag that is critical for crystal packing is indicated by the red dotted circle. The view focused on the single AcrIF9 molecule in the asymmetric unit is provided on the right side of the panel for a better view of crystal packing.

The type I CRISPR‐Cas system forms RNA‐guided multi‐subunit cascade complexes. Cas3 (trans‐acting nuclease) is involved in this system to cleave the target DNA (Fig. 1A). The type I CRISPR‐Cas system is divided into six subtypes, I‐A to I‐F, based on the subunit composition in the cascade complex [28]. Because classification of Acr depends on the target CRISPR‐Cas systems, AcrI proteins, which target the type I CRISPR‐Cas system, can be divided into six families, AcrIA to AcrIF. Among these families, the inhibitory mechanism of Acr has been intensively analyzed with structural studies of the AcrIF family. Previous studies have shown that the AcrIF family inhibits the type I‐F CRISPR‐Cas system in two different ways: (a) directly binding to cascade complex proteins and blocking the target DNA interaction (e.g., AcrIF1 [29], AcrIF2 [17], and AcrIF10 [30]) or (b) directly binding to the Cas3 helicase/nuclease protein and inhibiting Cas3 interactions to target DNA (e.g., AcrIF3 [31]; Fig. 1A). Although diverse structures and mechanisms of several Acr have been revealed, the inhibitory mechanism of the AcrIF9 family has not been identified due to limited structural information. Thus, to understand the molecular basis underlying AcrIF9 anti‐CRISPR function, we purified AcrIF9 using two‐step chromatography, affinity chromatography and SEC. According to SEC, the protein was eluted around 19 mL of the SEC column, indicating that AcrIF9 exists as a monomer in solution (Fig. 1B). Although various AcrI families can function in monomeric form, previous structural and biochemical studies have shown that AcrI families, sometimes, form homodimers in solution [16, 31, 32]. Given the stoichiometric diversity of the AcrI families, we used MALS to determine the absolute molecular mass of AcrIF9 in solution, which was 11.2 kDa (8.2% fitting error) with 1.002 polydispersity (Fig. 1C). Because the theoretically calculated molecular weight of monomeric AcrIF9 with the C‐terminal histidine tag was 9.8 kDa, the peak may be attributable to monomeric AcrIF9. These SEC and MALS experiments indicate that AcrIF9 exists in a monomeric state in solution.

With no known structural homologues available in the PDB database, we were unable to solve the phasing problem by molecular replacement. However, the phase was able to be obtained using ARCIMBOLDO_BORGES *ab initio* phasing software, which can use small helix and sheet fragments available in the PDB for *ab initio* phasing. The final 1.2 Å structure was refined to *R*_work_ = 19.4% and *R*_free_ = 20.5%. The diffraction data and refinement statistics for AcrIF9 are summarized in Table 1. The crystal belongs to space group *P2_1_2_1_2_1_* with one molecule present in the asymmetric unit (Fig. 1D). The final model contains the complete sequences of AcrIF9 (from M1 to Q68) with six C‐terminal histidine residues and extra leucine and glutamic acid residues (LE) from the expression construct (Fig. 1D). The structure of AcrIF9 is composed of four antiparallel β‐sheets (S1–S4) surrounding one α‐helix (H1; Fig. 1D). Detailed topology analysis indicated that the fold of AcrIF9 is constructed with two antiparallel β‐sheets connected by one α‐helix in the middle (Fig. 1E). Crystallographic packing analysis showed that the uncleaved C‐terminal six‐histidine tag was critical for crystal packing by interacting with neighboring molecules (Fig. 1F). We failed to obtain the AcrIF9 protein crystal whose C‐terminal six‐histidine tag was removed, which may be due to the role of the tag in crystal packing.

**Table 1:** Data collection and refinement statistics.

Data collection	
Space group	*P2_1_2_1_2_1_*
Unit cell parameter *a*, *b*, *c* (Å)	
*a*, *b*, *c* (Å)	*a* = 27.23, *b* = 31.35, *c* = 80.70
α, β, γ (°)	α = 90, β = 90, γ = 90
Resolution range (Å)^a^	29.22–1.21
Total reflections	271 001
Unique reflections	21 825
Multiplicity	12.4 (11.19)
Completeness (%)^a^	99.97 (99.77)
Mean *I*/σ(*I*)^a^	17.0 (1.0)
*R*_merge_ (%)^a^, ^b^	7.2 (23.4)
*R*_meas_ (%)	7.5 (24.5)
CC_1/2_	9.3 (5.1)
Wilson *B*‐factor (Å^2^)	15.21
Refinement	
Resolution range (Å)	29.22–1.21
Reflections	21 812
*R*_work_ (%)	18.82 (41.11)
*R*_free_ (%)	19.54 (49.88)
No. of protein in the asymmetric unit	1
No. of nonhydrogen atoms	763
Protein	659
Solvent	104
Average *B*‐factor values (Å^2^)	19.3
Protein	16.84
Solvent	28.22
Ramachandran plot:	
Favored/allowed/outliers (%)	98.65/1.35/0
Rotamer outliers (%)	0
Clashscore	0.77
RMSD bonds (Å)/angles (°)	0.005/0.799

^a^ Values for the outermost resolution shell in parentheses.

^b^ *R*_merge_ = Σ*_h_* Σ*_i_* |*I*(*h*)*_i_* − <*I*(*h*)>|/Σ*_h_* Σ*_i_* *I*(*h*)*_i_*, where *I*(*h*) is the observed intensity of reflection *h*, and <*I*(*h*)> is the average intensity obtained from multiple measurements.

### Structural comparison with the cryo‐EM structure of cascade‐complexed AcrIF9

**Fig. 2:**
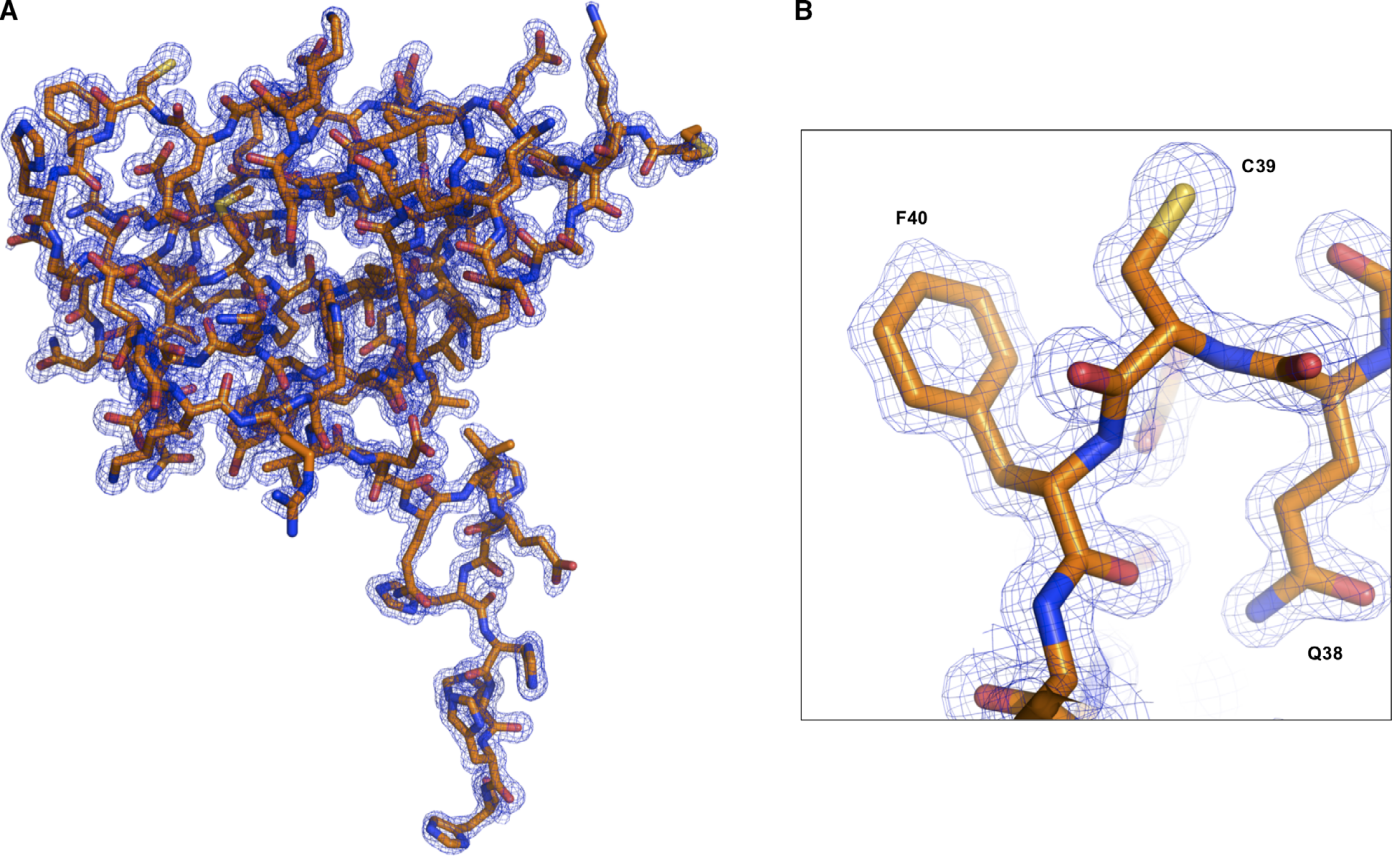
High‐resolution crystal structure of AcrIF9. (A) Quality of the electron density map for every atom in the structure. The 2Fo‐Fc density map contoured at the 1σ level is shown. (B) A magnified map around the representative region to show the quality of the structure. The region contains F40, and the hole in the center of the phenyl rings is visible in the structure

**Fig. 3:**
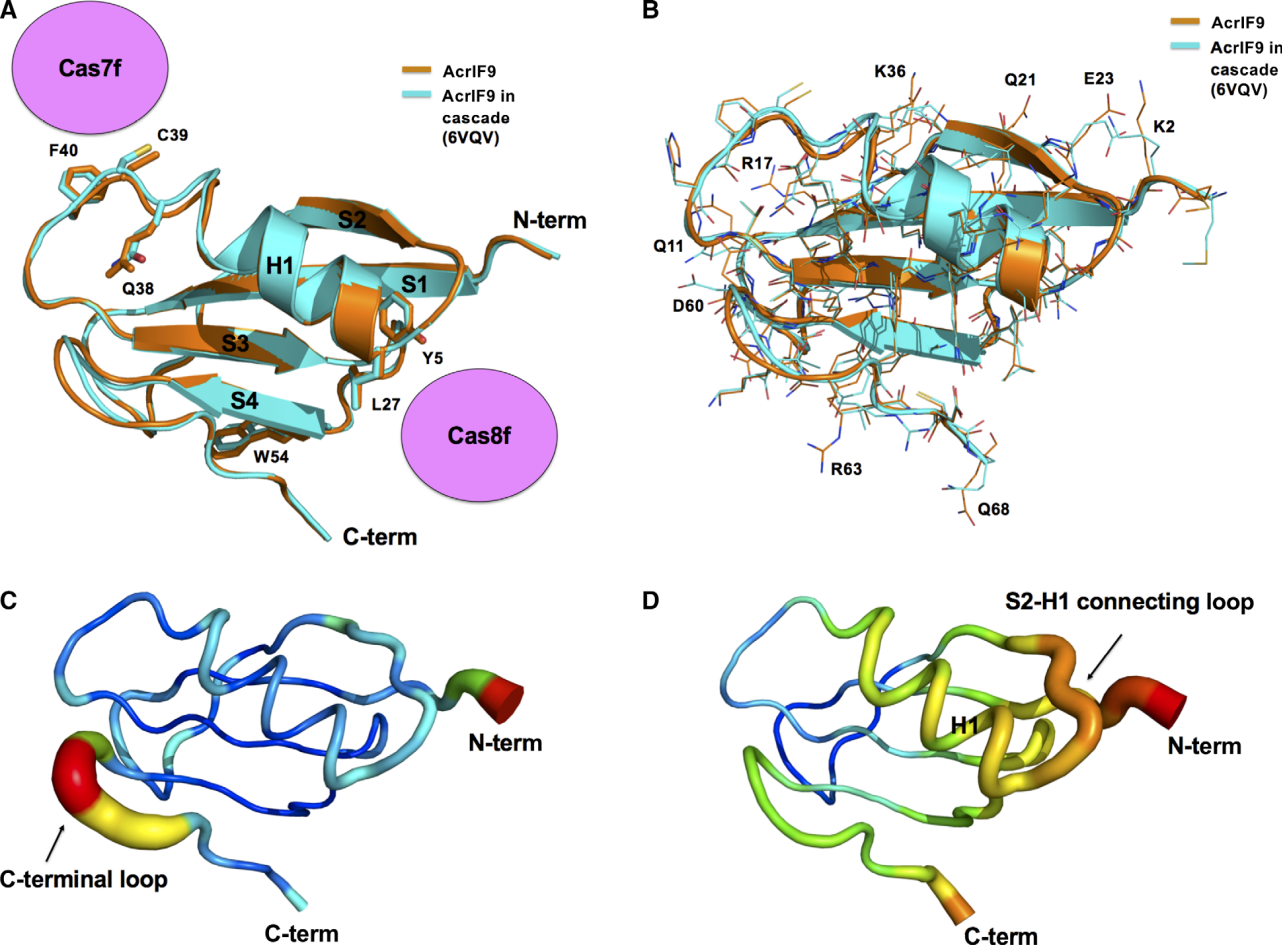
Structural comparison with the cryo‐EM structure of cascade‐complexed AcrIF9. (A) Superimposition of the current crystal structure of AcrIF9 (orange color) on the cryo‐EM structure of cascade‐complexed AcrIF9 (cyan color). The components of the cascade complex (Cas7f and Cas8f) are shown at the AcrIF9‐binding regions. The residues involved in interactions with Cas7f and Cas8f are labeled. (B) Superimposition of the current crystal structure of AcrIF9 (orange color) with the cryo‐EM structure of cascade‐complexed AcrIF9 (cyan color) to compare structural details focused on the positions of side chains. The residues with differing side chain structures are labeled. (C, D) *B*‐factor distribution of the crystal structure of AcrIF9 (C) and the Cryo‐EM structure of cascade‐complexed AcrIF9 (D). The structures are presented in a putty representation and rainbow‐colored from red to violet in *B*‐factor value order.

During our analysis of the AcrIF9 structure with further biochemical studies, Zhang *et al*. [18] released the cryo‐EM structure of AcrIF9 associated with the cascade complex. Because the advantage of our crystal structure was accuracy with extremely high resolution, we compared our structure with newly reported cryo‐EM structure. The 1.2 Å high‐resolution crystal structure of our AcrIF9 was highly ordered, and it was easy to see every atom in the electron density map (Fig. 2A). Even the hole in the center of phenyl rings (e.g., F40) was visible in our structure (Fig. 2B). A structural comparison with the cryo‐EM structure of cascade‐complexed AcrIF9 by pairwise superimposition showed that the overall structure was almost identical with a RMSD value of 0.5 Å; only the locations of a few loops, including the H1‐S3 connecting loop and the C‐terminal loop, did not align perfectly (Fig. 3A). The cryo‐EM structure showed that the inhibitory mechanism of AcrIF9 resulted from its direct binding to the cascade spiral backbone, particularly Cas7f and Cas8f, to prevent DNA binding. Because Y5 and L27 as well as Q38, C39, and F40 of AcrIF9 were involved in the interactions with Cas7f and Cas8f, respectively, we analyzed the cascade‐binding region of AcrIF9 to see whether any structural changes occurred during the process. This analysis showed that all side chains of Y5, L27, Q38, C39, and F40 from our crystal structure were exactly same as those from the cryo‐EM structure, indicating that the structure of AcrIF9 does not change when it binds to the cascade complex for inhibition (Fig. 3A). Although identical structures of cascade complex‐binding residues on AcrIF9 were detected, the locations of the side chains from many residues, especially those on the surface such as K2, Q11, R17, Q21, E23, K36, D60, R63, and Q68, were not identical, indicating that those surface residues have dynamic properties (Fig. 3B).

*B*‐factor analysis indicated that our high‐resolution crystal structure was rigid with a low *B*‐factor (average of 19.30 Å^2^), while the cryo‐EM structure was relatively less rigid with a higher *B*‐factor (average of 75.73 Å^2^; Fig. 3C,D). Interestingly, the highest *B*‐factor area in the crystal structure was the C‐terminal loop right next to S4 (Fig. 3C) while the highest *B*‐factor areas in the cryo‐EM structure were H1 and the S2‐H1 connecting loop (Fig. 3D). These findings indicate that AcrIF9 has a flexible C‐terminal loop and changes its structural properties after binding to the cascade complex. The flexible C‐terminal loop becomes rigid, and H1 along with the S2‐H1 connecting loop becomes less rigid after target protein binding.

### AcrIF9 directly binds to DNA as well as cascade complex proteins

**Fig. 4:**
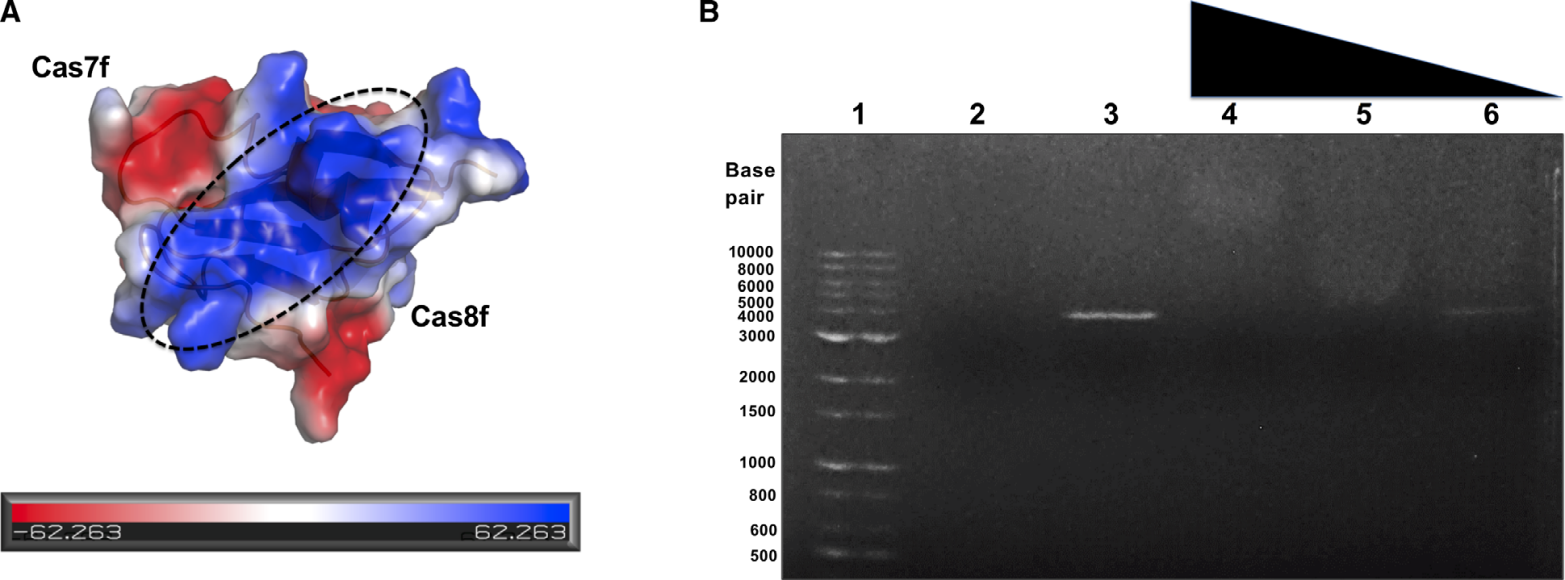
DNA‐binding capability of AcrIF9. (A) Surface electrostatic potential of AcrIF9. The respective surface electrostatic distributions are represented. The scale ranges from −6.2 kT/e (red) to 6.2 kT/e (blue). The black dashed circle indicates the tentative DNA‐binding region. (B) DNA‐binding activity assay by agarose gel shift of linearized plasmid DNA. Lane 1 contains the DNA size marker. Lane 2 contains 200 µm of AcrIF9 alone. Lane 3 contains linearized plasmid DNA alone. Lanes 4–6 contain DNA with 200 µm AcrIF9 (4), 100 µm AcrIF9 (5), and 20 µm AcrIF9 (6). The descending triangle indicates the gradient of protein concentration added.

The charge distributions and surface features were analyzed by calculating the surface electrostatic potential. This analysis showed that AcrIF9 contains a highly positively charged cleft between the Cas7f‐ and Cas8f‐binding regions (Fig. 4A). Based on this observation, we speculated that AcrIF9 may bind to negatively charged DNA and cascade proteins (Cas7f and Cas8f). According to a structural homology search using the Dali server [33], Cas3 (PDB id: 5B7I) [31] and Cas2 (PDB id: 4P6I) [34] were selected as structural homologues with AcrIF9 even though the top hits in order were antitoxin Dmd (PDB id: 5I8J) [35], PURS protein (PDB id: 1VQ3) [36], and insecticidal protein (PDB id: 5V3S) [37] (Table 2). Because Cas3 and Cas2 are nucleases/helicases, which are involved with DNA binding, structural homologue AcrIF9 also functions in binding to DNA. This structural homologue analysis also supports our idea that AcrIF9 may have the ability to bind to DNA. Finally, we performed a direct DNA‐binding test using an agarose gel shift assay. As indicated in Fig. 4B, linearized plasmid DNA was shifted up by adding AcrIF9 in a concentration‐dependent manner, indicating that AcrIF9 directly binds to DNA. It has been revealed that AcrIF9 inhibits CRISPR‐Cas systems by binding to the spiral backbone of CRISPR to prevent further DNA cleavage by Cas3. If AcrIF9 binds to DNA when it binds to CRISPR, AcrIF9 may highjack the targeted DNA by binding to the CRISPR complex. Our structural comparison with the cryo‐EM structure of cascade‐complexed AcrIF9 revealed that the Cas7f‐ and Cas8f‐binding regions of AcrIF9 were rigid in conformation with or without cascade proteins, whereas the S2‐H1 connecting loop and H1 of AcrIF9 became less rigid after binding to the cascade complex. Because the S2‐H1 connecting loop and H1 region are tentative DNA‐binding regions featuring highly positive charges, this AcrIF9 lack of rigidness after binding to the cascade complex may be important for further DNA recognition and subsequent inhibition of CRISPR‐Cas systems. Future biochemical and structural studies are needed to elucidate the meaning of the DNA‐binding capability of AcrIF9 during CRISPR‐Cas system inhibition.

**Table 2:** Structural similarity search using the Dali server [38].

Proteins (accession numbers)	*Z*‐score	RMSD (Å)	Identity (%)	References
Antitoxin Dmd (5I8J)	5.3	2.7	5	[35]
PURS (1VQ3)	3.9	2.4	6	[36]
Insecticidal protein (5V3S)	3.6	4.3	14	[37]
Cas3 (5B7I)	3.4	4.7	7	[31]
Cas2 (4P6I)	3.3	4.3	2	[34]

## Author contributions

HHP designed and supervised the project. GEK and SYL collected the data. HHP and GEK wrote the manuscript. All authors discussed the results and commented on and approved the manuscript.

## Conflict of interest

The authors declare no conflict of interest.

## Data Availability

The coordinates and structure factors have been deposited into the Research Collaboratory for Structural Bioinformatics (RCSB) Protein Data Bank (PDB). The PDB ID code is 7CHR.
